# Serum levels of receptor-interacting protein kinase-3 in patients with COVID-19

**DOI:** 10.1186/s13054-020-03209-6

**Published:** 2020-08-04

**Authors:** Hideta Nakamura, Takeshi Kinjo, Wakako Arakaki, Kazuya Miyagi, Masao Tateyama, Jiro Fujita

**Affiliations:** Department of Infectious, Respiratory and Digestive Medicine, Graduate School of Medicine, University of the Ryukyus, Okinawa, Japan

**Keywords:** COVID-19, Acute respiratory distress syndrome, Necroptosis, Receptor-interacting kinase 3

Dear Editor:

Patients with coronavirus disease 2019 (COVID-19) can develop acute respiratory distress syndrome (ARDS), which has been linked to poor prognosis and is a major contributor to patient death [1]. A better understanding of the pathophysiology of COVID-19-related ARDS would benefit early, precise treatment.

Cell death plays a major role in ARDS pathogenesis. While apoptosis in acute lung injury is well studied, newly identified cell death signaling has drawn attention as a potential mediator of ARDS [2]. Necroptosis, a caspase-independent form of necrosis involving receptor-interacting kinase 3 (RIPK-3), has been implicated in ARDS development with sepsis and trauma [3]. Since this highly regulated cell death signaling leads to rupture of the plasma membrane and release of damage-associated molecular patterns [4], necroptosis may be a therapeutic target for ARDS. However, the relationship between necroptosis and COVID-19-induced ARDS remains unclear.

Here, we describe serum RIPK-3 levels in COVID-19 patients measured on the first day of hospitalization. Patients were recruited from March 1 to May 30, 2020, and diagnosed as “severe” if any of the following conditions were met [5]: (1) respiratory rate > 30 breaths/min, (2) saturation of peripheral oxygen < 93% in ambient air, (3) ratio of arterial partial pressure of oxygen to the fraction of inspired oxygen < 300 mmHg, or (4) lung infiltrates > 50% within 24–48 h. Blood samples were centrifuged within 30 min and refrigerated at 4 °C, and plasma aliquots were frozen within 12 h. RIPK-3 levels were measured using an enzyme-linked immunosorbent assay (Wuhan Huamei Biotech, Wuhan, China).

This observational study enrolled 16 COVID-19 patients (11 males, 68.8%) (Table 1). Confirmation of severe acute respiratory syndrome coronavirus 2 (SARS-CoV-2) infection was by real-time reverse transcription polymerase chain reaction of nasopharyngeal swabs. Patients’ median age was 55 years (interquartile range [IQR] 40.5–71.5 years), and the median duration from symptom onset to hospitalization was 7 days (3.25–9 days). On admission, 14 patients (87.5%) were confirmed to have COVID-19 pneumonia by chest computed tomography, 10 patients were diagnosed with severe COVID-19 and ARDS, and 6 patients were diagnosed as mild. While hospitalized, the antiviral drug favipiravir was administrated to 11 patients (68.8%) in the context of a clinical trial, whereas azithromycin (*n* = 11, 68.8%), nafamostat (*n* = 12, 75%), and tocilizumab (*n* = 7, 43.7%) were commenced as off-label use. The median levels of serum RIPK-3 were significantly higher in severe COVID-19 cases than in mild cases (483.5 pg/mL, IQR 329.6–867.7 pg/mL vs. 139.9 pg/mL, IQR 95.37–286.8 pg/mL, *p* = 0.0075) (Fig. 1). Fifteen patients recovered and were discharged, whereas three patients in the severe group were intubated due to severe acute respiratory failure and one of these patients died.

**Table 1:** Clinical characteristics of patients (*n* = 16)

Male	11 (68.8%)
Median age, years (IQR)	55 (40.5–71.5)
Median duration from onset of symptoms to hospitalization, days (IQR)	7 (3.25–9)
Underlying disease	
Diabetes mellitus	4 (25%)
Hypertension	2 (12.5%)
Heart disease	2 (12.5%)
Treatment	
Azithromycin	11 (68.8%)
Favipiravir	11 (68.8%)
Nafamostat	12 (75%)
Tocilizumab	7 (43.7%)
Disease severity	
Mild	6 (37.5%)
Severe	10 (62.5%)
PaO_2_/FiO_2_ ratio	
> 350	6 (37.5%)
200–300	5 (31.25%)
150–200	3 (18.75%)
< 150	2 (12.5%)

*IQR* interquartile range, *PaO*_*2*_*/FiO*_*2*_*ratio* ratio of arterial partial pressure of oxygen to the fraction of inspired oxygen

**Fig. 1:**
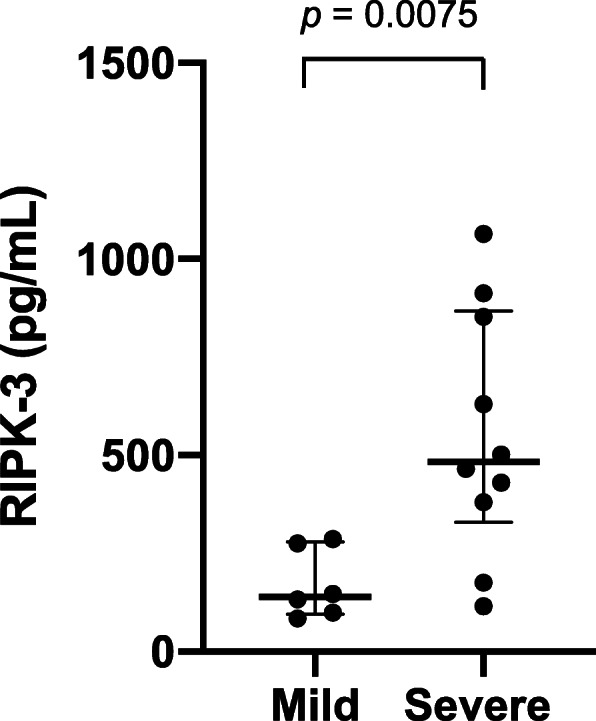
Serum levels of receptor-interacting kinase 3 (RIPK-3) in 16 patients with COVID-19. Serum RIPK-3 levels were measured by the enzyme-linked immunosorbent assay in patients with mild (*n* = 6) or severe (*n* = 10) COVID-19. For each dataset, the horizontal bars represent the median and interquartile range. Statistics were analyzed using Prism (GraphPad Software, CA, USA); *p* < 0.05 was considered significant

This is the first study to analyze RIPK-3 in COVID-19 patients. The higher serum RIPK-3 levels in severe patients suggest that RIPK-3-mediated signaling, such as necroptosis, might be involved in the development of acute lung injury associated with COVID-19 pneumonia. Siempos et al. reported plasma RIPK-3 levels were significantly higher in ARDS patients compared to those of non-ARDS patients [6]. Shashaty et al. demonstrated that among patients with sepsis or trauma, the change in plasma RIPK-3 levels 48 h after admission was independently associated with ARDS [3]. Because RIPK-3 mediates not only necroptosis but also other inflammatory pathways [2], the elevation of RIPK-3 does not directly indicate the execution of necroptosis. To confirm the role of RIPK-3 in COVID-19-ARDS patients, further studies are needed including a larger number of participants and histological evaluation of lung tissues, especially since RIPK-3-mediated necroptosis could be a potential therapeutic target for COVID-19-related ARDS.

## Data Availability

Full de-identified data of the analyses are available upon request sent to the corresponding author.

## Abbreviations

COVID-19: Coronavirus disease 2019
ARDS: Acute respiratory distress syndrome
RIPK1/3: Receptor-interacting kinase 1 and 3 (RIPK1/3)
IQR: Interquartile range
SARS-CoV-2: Severe acute respiratory syndrome coronavirus 2
